# Raccoons (*Procyon lotor*) as Sentinels of Trace Element Contamination and Physiological Effects of Exposure to Coal Fly Ash

**DOI:** 10.1007/s00244-016-0340-2

**Published:** 2016-12-08

**Authors:** Felipe Hernández, Ricki E. Oldenkamp, Sarah Webster, James C. Beasley, Lisa L. Farina, Samantha M. Wisely

**Affiliations:** 1 School of Natural Resources and Environment, University of Florida, 103 Black Hall, PO Box 116455, Gainesville, FL 32611 USA; 2 Department of Wildlife Ecology and Conservation, University of Florida, 110 Newins-Ziegler Hall, PO Box 110430, Gainesville, FL 32611 USA; 3 Savannah River Ecology Laboratory, University of Georgia, PO Drawer E, Aiken, SC 29802 USA; 4 Warnell School of Forestry and Natural Resources, University of Georgia, 180 E Green Street, Athens, GA 30602 USA; 5 Department of Infectious Diseases and Pathology, College of Veterinary Medicine, University of Florida, 2015 SW 16th Avenue, Gainesville, FL 32608 USA

## Abstract

Anthropogenic pollutants disrupt global biodiversity, and terrestrial sentinels of pollution can provide a warning system for ecosystem-wide contamination. This study sought to assess whether raccoons (*Procyon lotor*) are sentinels of local exposure to trace element contaminants at a coal fly ash site and whether exposure resulted in health impairment or changes in the intestinal helminth communities. We compared trace element accumulation and the impact on health responses and intestinal helminth communities of raccoons inhabiting contaminated and reference sites of the U.S. Department of Energy’s Savannah River Site (South Carolina, USA). Data on morphometry, hematology, histopathology, helminth community and abundance, and liver trace element burdens were collected from 15 raccoons captured adjacent to a coal fly ash basin and 11 raccoons from a comparable uncontaminated site nearby. Of eight trace elements analyzed, Cu, As, Se, and Pb were elevated in raccoons from the contaminated site. Raccoons from the contaminated site harbored higher helminth abundance than animals from the reference site and that abundance was positively associated with increased Cu concentrations. While we found changes in hematology associated with increased Se exposure, we did not find physiological or histological changes associated with higher levels of contaminants. Our results suggest that raccoons and their intestinal helminths act as sentinels of trace elements in the environment associated with coal fly ash contamination.

**Electronic supplementary material:**

The online version of this article (doi:10.1007/s00244-016-0340-2) contains supplementary material, which is available to authorized users.

Human-driven environmental factors have rapidly altered ecosystem composition and function on a global scale. Drivers of change include deforestation, hunting, introduction of invasive species, pathogen spread, and pollution (Hoffer and East 2012; Vitousek et al. 1997). Chemical contamination is a concern for ecosystem health and biodiversity loss, but compared with other anthropogenic stressors, the impact of pollutants in free-ranging wildlife populations has been less well-studied because responses are difficult to assess in the field (Hoffer and East 2012).

Among anthropogenic sources of contamination, combustion of coal for electrical power generation has resulted in substantial waste pollution, particularly in the form of fly ash (Rowe et al. 2002). Coal fly ash contains multiple trace elements, including arsenic (As), cadmium (Cd), chromium (Cr), copper (Cu), lead (Pb), selenium (Se), and mercury (Hg) (Patra et al. 2012; Rowe et al. 2002), which are released during combustion and introduced into the environment when accumulated ash is placed in settling ponds. More than 130 m tons of coal ash are generated each year in the United States, and federal regulations for its handling and disposal have recently been approved (Johnson 2009). Large ash contamination events, such as in Kingston, Tennessee, in December 2008 have raised public awareness about coal ash storage, deposition, and contamination, and their potential environmental and health impacts (Ruhl et al. 2009).

Coal ash toxicants, such as As, Cd, Pb, and Hg, affect multiple physiological systems in exposed animals, including the central nervous, hematopoietic, cardiovascular, gastrointestinal, urinary, and reproductive systems (Chmielnicka et al. 1989; Domingo 1994; Hughes et al. 2011). High levels of essential elements, such as Cr, Cu, Se, and zinc (Zn) also may impact reproductive, gastrointestinal, and hematopoietic systems (Domingo 1994; Eisler 1998; Ohlendorf and Fleming 1988). Numerous studies have found increased accumulation of trace elements and adverse health effects of coal ash in aquatic habitats; far fewer studies have focused on the impact to terrestrial systems (Rowe et al. 2002).

The effects of contaminants on biological systems have been assessed traditionally by using sentinels of pollution exposure. Sentinels are species that accumulate pollutants in tissues or display obvious signs of developmental and physiological impairment and thereby provide a baseline for understanding contaminant risk in a community or ecosystem (Beeby 2001). Sentinels have been used extensively to understand contamination of aquatic ecosystems. Several aquatic or semiaquatic guilds are commonly used, including predatory species that feed in aquatic food chains, such as fish, piscivorous birds, and mammals. These species accumulate trace elements quickly (Fisk et al. 2005; Mason and Wren 2001; Scheuhammer et al. 2007), yet rarely exhibit marked physiological abnormalities. Alternatively, aquatic amphibians and larval fish exhibit developmental abnormalities at relatively low concentrations of trace elements (Rowe et al. 1996). Previous studies investigated contaminant uptake in riparian- and aerial-feeding wildlife (Meyer et al. 2015) and more recently terrestrial mammals (Dainowski et al. 2015; Kalisinska et al. 2009; Millán et al. 2008; Naccari et al. 2013).

Endoparasites have been postulated as sentinels of ecosystem health and function (Marcogliese 2005; Marcogliese and Pietrock 2011). As a ubiquitous component of complex food webs, intestinal endoparasites may signal subtle but important changes to the functioning of these systems. Stressors, such as environmental pollutants, have been shown to reduce species richness of parasites via toxic effects to the parasite (MacKenzie 1999; Marcogliese 2004; Minguez et al. 2011). Also, parasitic species richness may decrease indirectly as a result of decreased intermediate host density (Lafferty 1997; Marcogliese 2005). Alternatively, increases in parasite abundance or species richness have been observed in hosts from contaminated systems due to suppression of the host immune system (MacKenzie 1999; Marcogliese and Pietrock 2011; Poulin 1992). Most investigations incorporating parasitology of host species as sentinels of pollution and environmental stress have focused on vertebrates in aquatic ecosystems; little is known about the effects of contaminants on parasites in terrestrial ecosystems.

Among terrestrial mammals, raccoons (*Procyon lotor*) are considered a sentinel species of environmental hazards (Gaines et al. 2000, 2002). Raccoons are omnivorous generalists that accumulate pollutants from eating contaminated terrestrial and aquatic resources (Bigler et al. 1975; Herbert and Peterle 1990; Lord et al. 2002). Raccoons are abundant, widely distributed, highly adapted to human-disturbed areas, and have small home ranges in proportion to body mass (Beasley et al. 2007, 2011; Gaines et al. 2000; Herbert and Peterle 1990). Raccoon contaminant uptake, therefore, is likely to represent local exposure (Burger et al. 2000; Gaines et al. 2000). Raccoons accumulate trace elements, such as As, Cd, Pb, Hg, and Se, but adverse health effects are less evident even in the face of high accumulation in tissues (Burger et al. 2002; Clark et al. 1989; Lord et al. 2002; Souza et al. 2013) (Table 1). Because raccoons are the definitive host of a large assemblage of intestinal helminths, including nematodes, cestodes, and acanthocephalans (Cole and Shoop 1987; Munscher 2006; Yabsley and Noblet 1999), they make an excellent study system for understanding the role of endoparasites and their host species as sentinels of sources of contamination, such as coal fly ash, in terrestrial ecosystems.

**Table 1:** Trace element concentrations (mg/kg) in raccoons, reported as originally published in comparative studies between contaminated and reference sites

Location	Tissue	Trace element	Concentration	*p*	Reference
California					Clark et al. (1989)
Contaminated site (*n* = 8)	Liver	Se	19.9^a^	<0.001	
Reference site (*n* = 4)			1.69^a^		
South Carolina					Burger et al. (2002)
Contaminated site (*n* = 13)	Kidney	As	0.68 ± 0.07^b^	<0.05	
Reference site (*n* = 12)			0.1 ± 0.01^b^		
	Liver	Cd	0.5 ± 0.09^b^	<0.05	
			0.94 ± 0.10^b^		
	Liver	Cr	0.45 ± 0.06^b^	<0.05	
			0.32 ± 0.02^b^		
	Liver	Cu	11.4 ± 1.17^b^		
			14.6 ± 1.17^b^		
	Liver	Pb	0.33 ± 0.09^b^		
			0.45 ± 0.10^b^		
	Liver	Se	3.88 ± 0.60^b^		
			2.54 ± 0.13^b^		
South Carolina					Lord et al. (2002)
Contaminated site (*n* = 13)	Liver	Hg	0.66^c^		
Reference site (*n* = 12)			1.31^c^		
Tennessee					Souza et al. (2013)
Contaminated site (*n* = 10)	Hair	As	0.405^d^	<0.05	
Reference site (*n* = 10)			0.092^d^		
	Hair	Cr	0.493^d^		
			0.227^d^		
	Liver	Cu	9.35^d^		
			7.75^d^		
	Liver	Hg	0.245^d^		
			0.122^d^		
	Liver	Se	1.05^d^		
			0.89^d^		
	Liver	Zn	39.85^d^		
			40.9^d^		

^a^Geometric mean; concentrations are mg/kg dry-weight

^b^Arithmetic mean (±SE); concentrations are mg/kg wet-weight

^c^Geometric mean; concentrations are mg/kg wet-weight

^d^Median; concentrations are mg/kg wet-weight

This study explores the role of raccoons as biosentinels of both trace element exposure and effects on their intestinal parasite community assemblage as a result of exposure to coal fly ash at the U.S. Department of Energy’s Savannah River Site (SRS). SRS is a former nuclear weapons production facility characterized by the occurrence of industrial areas, including a coal-powered electricity generation facility that released industrial contaminants into the surrounding environment (White and Gaines 2000). The objectives of our study were to test the hypotheses that: (1) raccoons inhabiting the coal fly-ash contaminated site would have higher concentrations of trace elements in target tissues (liver) compared with animals from an uncontaminated reference site; (2) raccoons with elevated levels of trace elements would exhibit altered health parameters (poor body condition as seen by morphometrics, and hematologic and histologic abnormalities), as a result of contaminant accumulation; and (3) intestinal helminth populations would respond positively to contaminants due to the suppressed immune function of the host. Alternatively, intestinal helminths could be depressed in species richness and abundance by environmental contamination with no suppression of the immune system. Our goal was to develop an integrative understanding of how anthropogenic perturbations in terrestrial ecosystems affect the interplay between raccoon health and parasite assemblages.

## Materials and Methods

### Study Area

SRS is located in west-central South Carolina, USA. The former nuclear production site encompasses 78,000 ha (778 km^2^) and has had restricted public access since 1951 (White and Gaines 2000). In 1972, the SRS was classified as the first U.S. National Environmental Research Park where the ecological impacts of anthropogenic manipulation of the environment could be studied (White and Gaines 2000). Approximately 90% of the SRS is undeveloped. The undeveloped area is comprised of managed pine timber production (54%), wetlands (23%), upland hardwood and mixed forest (11%), grasslands (9%), and upland scrub forest (3%) that are interspersed across the landscape (White and Gaines 2000). Between 1951 and 1988, industrial production activities on the SRS resulted in several releases of contaminants into the surrounding habitats on site. These perturbations included pollution with anthropogenic compounds, such as radionuclides, coal fly-ash runoff, and other trace elements into the surrounding ecosystems (White and Gaines 2000). As a result of restricted public access, the SRS has become a refuge for many wildlife species, including raccoons, which are known to occupy both contaminated and uncontaminated habitats on site (Gaines et al. 2002).

**Fig. 1:**
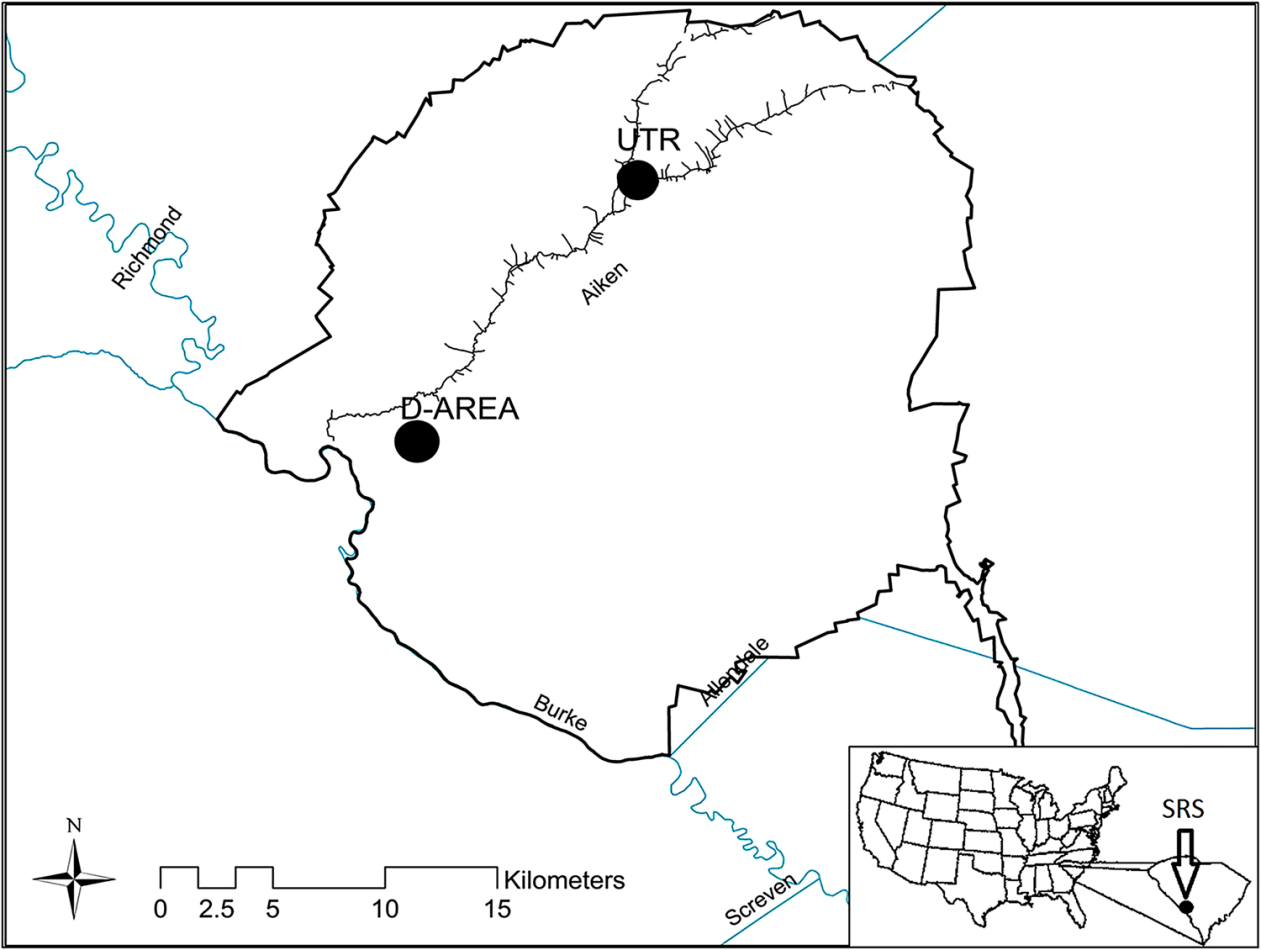
Map of the U.S. Department of Energy’s Savannah River Site (33.1°N, 81.3°W) shows the contaminated coal fly ash area (D-Area) and reference Upper Three Runs Creek (UTR) sites where raccoons were collected

For this study, two discrete areas within the SRS were selected to collect raccoons in August and December 2013: an area known to be contaminated with trace elements and a reference site that was uncontaminated (Fig. 1). The first location—the D-Area ash basins—was the site of a coal-fired power plant that deposited sluiced fly ash into a series of basins from 1953 to 2012 while the plant was operational (U.S. Department of Energy 2012). These basins drained into Beaver Dam Creak, a tributary of the Savannah River, and the surrounding wetlands (Bryan et al. 2012; Gaines et al. 2002). The basins and creek watershed have been shown to have increased levels of aluminum (Al), As, Cd, Cr, Cu, iron (Fe), Hg, manganese (Mn), nickel (Ni), Se, and Zn in the water and biota (Cherry et al. 1979; Rowe et al. 1996). Thus, raccoons were collected in the forested areas surrounding the basins as well as riparian habitats adjacent to Beaver Dam Creek.

Upper Three Runs Creek (UTR) was selected as the control site for this study and is a natural creek originating from a spring-fed aquifer system that has not been impacted by industrial activities on the SRS (Bowers 1997). Beaver are common in the area and numerous beaver ponds and Carolina bays exist in the vicinity of UTR. Thus, a combination of wetland and creek habitats as well as a mixture of mixed hardwood and pine forest existed on each site. The similar habitat composition between the two sites allowed us to assume similar food availability to raccoons across the study system. Although some sublethal effects have been observed in some prey species exposed to coal fly ash, common prey items of raccoons are present in the ash basin utilized in this study (Hopkins et al. 1998, 1999). The area of UTR where raccoons were trapped was approximately 16 km from the D-Area ash basins, eliminating the possibility of individual raccoons traveling between the two areas, as raccoon home ranges generally are <2–4 km^2^, although home ranges up to 10 km in diameter have been reported (Gehrt and Fritzell 1997). UTR, referred to hereafter as the reference site, has been used as an undisturbed reference site in several publications (Burger et al. 2002; Morse et al. 1980).

### General Approach

To evaluate whether raccoons were sentinels of trace element contamination, we tested individual parameters (age, site, sampling season and site*sampling season interaction) as predictors of elevated concentration of eight trace elements in the liver of raccoons collected in contaminated versus reference sites. For trace elements that exhibited differences among contaminated and reference sites, we considered them as predictor variables to assess changes in morphometry, hematology, and parasite burden of individual raccoons across contaminated and reference sites.

### Trapping and Necropsy Methods

In August and December 2013, raccoons were captured using steel wire box live traps (Tomahawk Live Trap, Hazelhurst, Wisconsin), baited with whole corn, fish meat, and/or plaster scent tabs. Traps were placed strategically along roads, water bodies, or in a grid within 500 m from the centroid of the reference site or the fly ash containment basins to target animals utilizing resources known to be uncontaminated or contaminated, respectively. After capture, animals were transported from the field to the Savannah River Ecology Lab animal care facility, where they were immobilized using Telazol (2.2 mg/kg IM) and subsequently euthanized via intracardiac injection of potassium chloride administered while the animal was under general anesthesia. All animal handling practices and euthanasia were performed in accordance with University of Georgia Animal Care and Use guidelines under protocol A2012 12-010-Y2-A3.

Twenty-six raccoons were trapped throughout the two trapping seasons, and sex and estimated age classes were recorded. Raccoons were grouped into three age classes: I (1–2 years), II (3–4 years), and III (5–6 years) based on tooth wear. We captured 11 raccoons in August [contaminated site *n* = 4 (all males); reference site *n* = 7 (6 males and 1 female)] and 15 raccoons in December [contaminated site *n* = 11 (5 males and 6 females); reference site *n* = 4 (all males)]. Seven raccoons were aged at 1–2 years (contaminated site *n* = 4; reference site *n* = 3), 12 aged at 3–4 years (contaminated site *n* = 7; reference site *n* = 5), and 7 aged at 5–6 years (contaminated site *n* = 4; reference site *n* = 3).

Raccoons were weighed and standard morphometric measurements were collected, from which body mass/nose-anus length ratio was calculated. Approximately 3 ml of blood was collected, two blood smears were made, and 2 ml of blood were transferred to ethylenediaminetetraacetic acid (EDTA) Vacutainer tubes (Becton, Dickinson and Co., Franklin Lakes, NJ). Tubes were refrigerated and shipped on ice overnight to the Georgia Veterinary Diagnostic and Investigational Laboratory (VDIL; University of Georgia, Tifton, GA) within 48 h of collection. Tissue samples were taken from the liver, spleen, kidneys, ileum, and colon and fixed in 10% neutral buffered formalin for histopathology analyses. Liver samples for trace element quantification were placed in Whirl–pak bags and frozen at −20 °C until analysis. The intestinal tract, including colon, was frozen and stored for helminth identification.

### Trace Element Analysis

Trace element [Cr, Ni, Cu, Zn, As, Se, Cd, Pb, uranium (U), and total mercury (THg)] analysis was conducted on all liver samples. Wet weights of liver samples were recorded and tissues subsequently freeze-dried and homogenized. For trace element analysis, approximately 250 mg of dry sample was used for microwave digestion (MARSX Xpress, CEM Corporation, Matthews, NC) with 10.0 ml trace metal-grade nitric acid (70% HNO_3_). After digestion, samples were brought to a final volume of 15.0 ml with Milli-Q (18 MΩ) water before analysis was performed by inductively coupled plasma mass spectroscopy (Nexlon 300X ICP-MS; Perkin Elmer, Norwalk, CT) according to the Quality Assurance/Quality Control (QA/QC) protocols outlined in EPA Method 6020A (USEPA 2007). Minimum detection limits (MDL; ppm) for each element were: Cr (0.54), Ni (0.84), Cu (0.67), Zn (5.15), As (0.13), Se (0.18), Cd (0.15), Pb (0.05), and U (0.09). Element concentrations lower than MDL were substituted by half of the corresponding MDL as standard procedure. Included in the digestion analysis for quality control purposes a certified reference material (TORT-3 lobster hepatopancreas; National Research Council, Ottawa, ON, Canada), a blank, and a digestion replicate were each run in conjunction with every 20 samples. For elements in certified reference materials, mean percent recoveries ranged from 85 to 105%.

For THg analysis subsamples of the freeze-dried and homogenized liver tissue (30–50 mg) were analyzed using a DMA 80 Dual Cell Mercury Analyzer (Milestone, Shelton, CT) according to U.S. Environmental Protection Agency (EPA) method 7473, which has an instrument detection limit (IDL) for this method of 0.01 nanograms (ng) of total mercury. For quality assurance, we included with each set of 10 samples a replicate, a blank, and two standard reference materials (SRM; TORT-2 lobster hepatopancreas and PACS-2 marine sediment, National Research Council of Canada, Ottawa, ON). Solid SRMs were used to calibrate the instrument. Method detection limits (MDLs; threefold the standard deviation of procedural blanks) averaged 0.0233 parts per billion (ppb) dry mass. Mean percent recoveries of THg for the SRMs TORT-2 and PACS-2 were 102.1 ± 1.6 and 103.8 ± 3.9, respectively. All THg concentrations were converted to parts per million (ppm) presented on a dry mass basis to be comparable to the other trace element concentrations.

### Hematology

Complete blood cell counts were conducted for 24 of 26 raccoons, excluding the other two animals in which blood samples were clotted. Anticoagulant (EDTA) blood tubes were processed for red blood cell (RBC) and total white blood cell (WBC) counts, mean corpuscular volume (MCV), hematocrit (HCT), mean corpuscular hemoglobin (MCH), mean corpuscular hemoglobin concentration (MCHC), hemoglobin (HGB), platelet counts, mean platelet volume (MVP), plasma protein, and reticulocyte and absolute reticulocyte counts by automation using the Advia 2120i Hematology System (Siemens Biomedical Solutions Diagnostics, Tarrytown, NY). Blood smears were stained with Wright’s (Romanosky) and Giemsa Stain (counter stain) procedure (Poly Scientific, Bay Shore, NY), and examined for cellular morphology, WBC differential cell counts, and blood parasites.

### Histopathology

Histopathologic analyses were conducted in liver, spleen, kidney, ileum, and colon samples collected from the 11 raccoons trapped in August 2013. These analyses were not conducted in raccoons from December 2013 because of logistical constraints. After fixation, sections of these tissues were processed routinely and embedded in paraffin. Sections that were 5-μm thick were stained with hematoxylin and eosin. Slides were examined by a single, blind observer (LF) for inflammation, fibrosis, degeneration/necrosis, pigment deposition, and other changes. A grading scheme was created and each tissue was graded subjectively for multiple parameters on a 0 (no changes), 1 (mild changes), 2 (moderate changes), and 3 (marked changes) scale for a total of 103 categorical variables. We included in the final analyses only 16 variables that were found to have substantial variation in the breadth of abnormalities and 3 binary categories (Table A.1; Online Resource 1).

### Helminth Sampling

Upon thawing, intestines were carefully opened along their length with scissors and contents were initially screened for helminths by hand. Intestines and digested material were rinsed through a 180-µm sieve to ensure all parasites were collected; the solid material left after rinsing was sorted again under a dissecting microscope. Parasites were preserved in absolute ethanol and identified to genus or species (when possible) using a compound microscope. Total number of helminths was determined for each raccoon using a dissecting scope.

### Data Analysis

Distributions of all variables were tested for normality (Shapiro–Wilkes test, *p* < 0.05), and all further statistical tests were conducted using log-transformed data. Based on a backward elimination procedure, stepwise regression models were used to test for differences in trace element exposure between raccoons from the contaminated and reference sites. Previous to their definitive inclusion in stepwise regression models, we tested for the univariate effect of site, age and sampling season on trace element accumulation of raccoons. We only included male data to test the univariate effect of age and sampling season because of the unbalanced sample size of sexes distributed among contaminated and reference sites. We broke down age by site and season to rule out the potential association of either site or season with animal age in the univariate analysis.

To determine the impact of trace element accumulation and sampling season on morphometry (body size variation—*PC1*), hematology (RBC, WBC, and platelet counts), and parasite burden (helminth abundances) of individual raccoons, we considered the spectrum of trace elements that were at higher concentrations in livers of raccoons from the contaminated site compared to the reference site as predictor variables. We used stepwise regression models with backward elimination, including morphometry, hematology, and parasitology responses in separate models. To test the impact of trace elements on raccoon morphometry and hematology, we considered all raccoons from contaminated and reference sites. Before the inclusion of trace element concentrations in the stepwise regression models, Pearson correlation coefficients were calculated to determine collinearity between the predictor variables. To reduce the number of variables analyzed, we conducted principal component analyses (PCA) independently on the morphometric dataset. Principal components that accounted for at least 70% of the total variance and whose eigenvalues were >1 were retained for interpretation and incorporation into multivariate regression models.

To test for relationships between the degree of histological lesions and trace element concentrations in raccoon livers from contaminated and reference sites in August 2013, we used multinomial logistic regression models only for 16 of the 103 categorical histological variables (16 variables which had substantial variation in degree of abnormalities). To test whether binary response variables (presence/absence of hepatic, ileum and colon granulomas, and blood parasites) correlated to contaminant exposure, simple logistic regression models were performed.

Nonmetric multidimensional scaling (NMDS) was used to evaluate visually the variation in community composition and abundance of intestinal helminth species harbored by all raccoons from contaminated and reference sites. We used abundance data for each helminth species from individual raccoons to calculate the Sorensen (Bray–Curtis) distance metric among raccoons from both sites. Two-dimensional analysis was chosen by minimizing stress, a goodness-of-fit measure of the mismatch between the rank-order of Euclidean distance in the ordination space, and the distance among species indicated by the Bray–Curtis dissimilarity matrix. Relationships between trace element concentrations and the ordination scores were graphically explored.

Stepwise regression models with backward elimination were used to test for the influence of trace element concentrations and sampling season on (non-log-transformed) parasite abundances of raccoons collected at both contaminated and reference sites. Because male and female raccoons have previously been found to have different parasite abundances (Cole and Shoop 1987; Kresta et al. 2009), and we had an unbalanced sample size between sexes in contaminated (9 males vs. 6 females) and reference (10 males vs. 1 female) sites, we only included male data in the stepwise regression models. In further stepwise regression models, we included WBC counts as an additional predictor of parasite abundance to test how biomarkers of immune response and trace element concentrations may influence parasite infestation in raccoons.

Statistical tests were conducted in the software R v. 2.15.2 (R Core Team 2012) and statistical significance was set at *α* = 0.05. NMDS ordination and vector fitting were conducted using the “ecodist” package in R (Goslee and Urban 2007).

## Results

### Concentrations of Trace Elements in Liver Tissue of Raccoons

We found no effect of age (neither considering all animals nor broken down by site or season) on trace element accumulation in raccoons (β_1_ coefficients and *t*-values of all univariate regression models associated with *p* > 0.05); thus, raccoons of all three age classes were pooled in stepwise regression models. As such, we only included site, sampling season and their interaction as predictors of trace element accumulation in raccoons. We found a significant influence of site and sampling season on the exposure of trace elements in raccoons. As, Se, and Pb were at higher concentrations in livers of raccoons inhabiting the contaminated site than in the reference site (Table 2). Only Ni was at higher concentrations in the livers of raccoons from the reference site than the contaminated site (Table 2). In addition, we found a significant relationship between the sampling season and exposure to Cr, Ni, As, and Pb; raccoons sampled in August had higher levels of these elements compared with animals sampled in December (Table 2). Additionally, we found a significant influence of the interaction site*sampling season on the exposure to Cu, As, and Pb in raccoons, with animals exhibiting higher liver concentrations of these trace elements in the contaminated site in August (Table 2). The main regression model for Cu was not statistically significant likely due to the significant interaction of site and season. The significant effect of site indicated an increase of Cu concentrations in raccoons from the contaminated compared with the reference site (Table 2). No correlation was detected between Cu*Se, Cu*Pb, As*Se, and Se*Pb, but significant associations between Cu*As and As*Pb were found (Table A.2; Online Resource 2).

**Table 2:** Mean (±SE) trace element concentrations measured in liver tissue of raccoons from contaminated (*n* = 15) and reference (*n* = 11) sites in the SRS (August and December 2013)

Trace element (mg/kg)	Sites		Model outputs					
	Contaminated	Reference	*R*_*adj*_^2^	*F*	*p*	β_1_	*t*-value	*p*
Cr	0.87 ± 0.15	1.36 ± 0.33	0.17	6.29	**0.02**	Season: −0.27	−2.51	**0.02**
Ni	0.51 ± 0.06	0.89 ± 0.17	0.58	12.58	**<0.001**	Site: 0.20	2.18	**0.04**
						Season: −0.20	−2.42	**0.02**
						Site: season: −0.20	−1.60	0.13
Cu	34.53 ± 4.24	29.24 ± 3.78	0.11	1.99	0.15	Site: −0.24	−2.35	**0.03**
						Season: −0.18	−1.85	0.08
						Site: season: 0.30	2.16	**0.04**
Zn	112.98 ± 5.83	106.02 ± 6.39						
As	0.34 ± 0.07	0.25 ± 0.05	0.50	9.25	**<0.001**	Site: −0.41	−3.94	**<0.001**
						Season: −0.50	−5.07	**<0.001**
						Site: season: 0.41	2.88	**0.009**
Se	8.41 ± 0.83	3.96 ± 0.34	0.57	17.56	**<0.001**	Site: −0.27	−4.52	**<0.001**
						Season: 0.11	1.88	0.07
Cd	1.63 ± 0.38	1.61 ± 0.45						
Pb	0.54 ± 0.29	0.24 ± 0.04	0.21	3.27	**0.04**	Site: −0.49	−2.70	**0.01**
						Season: −0.50	−3.01	**0.007**
						Site: season: 0.58	2.38	**0.03**
Hg	3.68 ± 0.77	3.03 ± 0.46						

Trace element concentrations lower than MDL were substituted by a half of the corresponding MDL before statistical analysis. Values in bold denote statistical significance (*p* < 0.05) of the stepwise regression models and their corresponding independent variables (site and season). Zn, Cd and Hg concentrations were not significantly related to site and/or season

### Morphometry

Mean (±SE) values for all morphometric measurements were reported for male and female raccoons captured in the contaminated and reference sites (Table A.3; Online Resource 3). For the morphometric PCA, the first principal component (*PC1*) accounted for 82% of the variance (eigenvalue 41.31); PC loadings (eigenvectors) for each morphometric measurement were reported (Table A.4; Online Resource 4). *PC1* was interpreted as the range of body size variation among sampled raccoons, supported by the inverse relationship of PC values to morphometric measurements. Neither exposure to trace elements nor sampling season were significantly related to *PC1* in raccoons in the final stepwise regression model.

### Hematology

RBC, total WBC, and platelets counts were significantly lower in raccoons from the contaminated site compared to the reference site, and each of these hematological values significantly decreased in raccoons with elevated levels of Se (Table 3). Neither exposure to Cu, As, and Pb nor sampling season were significantly related to RBC, total WBC, and platelet counts. Neither trace elements nor sampling season were significantly related to presence of blood parasites (microfilaria).

**Table 3:** Mean ± S.E. (range) of values of hematology and presence of blood parasites (microfilaria) of raccoons from contaminated and reference sites in the SRS (August and December 2013)

Hematology	Reference values (Mean ± SD)	Sites		*R*_*adj*_^2^	*F*	Model outputs		*t*-value	*p*
		Contaminated	Reference			*p*	β_1_		
*N*		13	11						
RBC (10^6^/µl)	8.74 ± 1.20	7.85 ± 0.39 (6.36–10.46)	8.36 ± 0.30 (6.86–10.12)	0.13	4.46	**0.05**	Se: −0.12	−2.11	**0.05**
Total WBC (10^3^/µl)	9.84 ± 4.09	11.78 ± 1.04 (5.4–18.1)	13.05 ± 1.03 (8.5–18.2)	0.30	5.92	**0.01**	Se: −0.35	−3.21	**0.004**
							Pb: 0.12	1.57	0.13
Platelets (10^3^/µl)	470 ± 160	443.69 ± 48.57 (171–648)	577.09 ± 43.62 (314–710)	0.20	6.58	**0.02**	Se: −0.42	−2.57	**0.02**

Blood parasites	Reference values (Mean ± SD)	Sites		*R*_*adj*_^2^	*F*	Model outputs		*z*-value	*p*
		Contaminated	Reference			*p*	β_1_		
*N*		15	11						
Microfilaria presence/absence		2 infected raccoons	8 infected raccoons				Cu: 11.06	1.68	0.09
							Se: −9.36	−1.81	0.07
							Season: −2.97	−1.66	0.10

Values in bold denote statistical significance (*p* < 0.05) of the stepwise regression models and their corresponding independent variables (trace element and season). Reference values of raccoon hematology published by Denver (2003)

### Histopathology

Relevant histological injuries detected in raccoons trapped in August 2013 were confined to 16 of the 103 variables (Table A.1; Online Resource 1). These injuries were defined as hepatic fibrosis, eosinophilic hepatic portal inflammation, hepatic granulomas, bile duct proliferation and pigment in hepatocytes; splenic lymphoid and reticuloendothelial hyperplasia; cellular infiltrates in intestinal lamina propria and submucosa, and intestinal granulomas. We tested nominal histological variables against the two trace elements, As and Se, which were at higher concentrations in livers of raccoons from the contaminated site compared to the reference site in August 2013. We did not find any association, however, between histological findings and As and Se (β_1_ coefficients and z-scores of all 16 multinomial logistic regression models had *p* > 0.05). For binary variables, we found no significant association of As and Se to the presence or absence of abnormalities and granulomas in liver, ileum, or colon in raccoons (β_1_ coefficients and *z*-values of all three logistic regression models had *p* > 0.05).

### Helminth Species

An average of 8.93 ± 2.32 parasites per host were detected in raccoons from the contaminated site, whereas 7.27 ± 2.77 parasites per host were detected in raccoons from the reference site. Among all raccoons, we found four helminth taxa that could be identified to species: two nematode species (*Placoconus lotoris* and *Physaloptera rara*), one cestode (*Atriotaenia procyonis*), and one acanthocephalan (*Macracanthorhynchus ingens*). In the contaminated site, we found one trematode but could only identify it to genus (*Isthmiophora* sp.); one raccoon had an unidentifiable strigeid trematode. Of the raccoons from the contaminated site, there was a mix of the four identified parasite species, with no individual having all four. *M. ingens* was the most abundant and prevalent helminth in both contaminated and reference sites, whereas *A. procyonis* was only present in raccoons from the contaminated site (Table 4). Raccoons from the reference site had a mix of *P. lotoris*, *P. rara*, and *M. ingens*, whereas *A. procyonis* was absent. None of the helminth species was found in all the infected raccoons.

**Table 4:** Mean ± S.E. (range) of values of helminth abundance (individual parasites per raccoon) of raccoons from contaminated and reference sites in the SRS (August and December 2013)

*Helminths* species	Sites		*R*_*adj*_^2^	*F*	Model outputs		*t*-value	*p*
	Contaminated	Reference			*p*	β_1_		
*N*	15	11						
*Placoconus lotoris*	0.73 ± 0.30 (0–4) (40%)	1.91 ± 1.27 (0–14) (36%)	0.08	2.64	0.12	Cu: 6.57	1.63	0.12
*Physaloptera rara*	1.60 ± 0.87 (0–13) (40%)	0.91 ± 0.28 (0–3) (64%)	0.24	6.64	**0.02**	Cu: 8.74	2.58	**0.02**
*Atriotaenia procyonis*	3.13 ± 1.92 (0–26) (20%)	0 (0%)						
*Macracanthorhynchus ingens*	3.47 ± 0.79 (0–10) (67%)	4.45 ± 1.57 (0–15) (73%)	0.26	4.09	**0.04**	Cu: 9.35	1.86	0.08
						Season: 4.15	2.33	**0.03**

Percentage of raccoons infected at each site (number of raccoons infected/total number of raccoons per site) is indicated in parentheses. Values in bold denote statistically significant (*p* < 0.05) stepwise regression models and their corresponding independent variables (Cu and season). Abundance of *Atriotaenia procyonis* was not significantly related to Cu concentrations and/or season

**Fig. 2:**
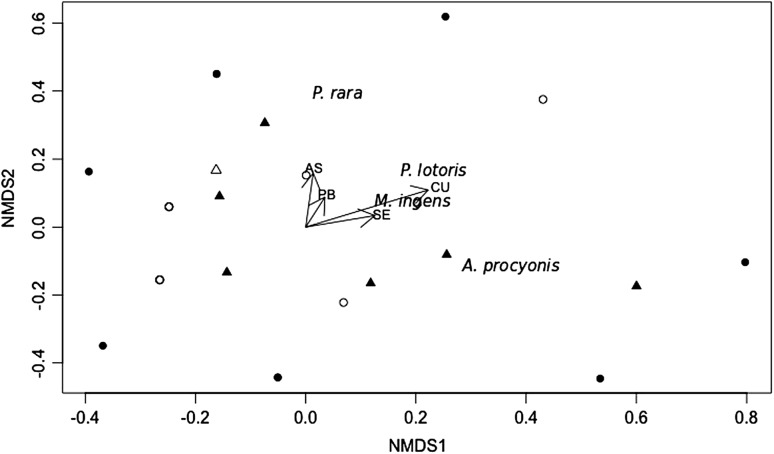
Nonmetric multidimensional scaling (NMDS) plot of intestinal helminth populations harbored by male (*black circles*, *n* = 9) and female (*black triangles*, *n* = 6) raccoons from contaminated site, and male (*white circles*, *n* = 10) and female (*white triangles*, *n* = 1) raccoons from reference site in the SRS (August and December 2013). Helminth species are indicated based on the species ordination scores. Proximity between helminth species indicates similar helminth abundances. *Black thick* radiating lines represent the relationships between trace element concentrations (Cu, As, Se, and Pb) and the ordination scores

NMDS two-dimensional ordination showed a minimum stress of 0.21 and accounted for 74% of the variance in the helminth abundance data. The ordination pattern showed that the helminth community composition differed between raccoons from contaminated and reference sites, but there was a larger variance in the helminth abundance in males from the contaminated site than the reference site (Fig. 2). *A. procyonis* was present in the contaminated site and absent at the reference site, but the abundance of *P. lotoris*, *P. rara*, and *M. ingens* was not different among sites (Table 4). Of all trace elements that were at higher concentrations in livers of raccoons from the contaminated site compared with the reference site, only the concentration of Cu was significantly related to an increase in *P. rara* (Table 4). *M. ingens* was more abundant in raccoons captured in December (Table 4). Cu liver concentrations were significantly related to the total abundance of helminths (model: *R*_*adj*_^2^ = 0.39, *F*_2,16_ = 6.76, *p* = 0.007; Cu: β_1_ 29.35, *t*-value 3.03, *p* = 0.008) in raccoons. Helminth total abundance was higher in raccoons captured in December than in August (Season: β_1_ 8.02, *t*-value 2.35, *p* = 0.03). WBC counts was not a significant predictor in any of the stepwise regression models that included helminth abundance (β_1_ coefficients and *z*-values of all models associated to *p* > 0.05).

## Discussion

Our results support the hypothesis that raccoons are sentinels of pollution by coal fly ash. Our study is the first to demonstrate increased accumulation of Cu and Pb concentrations in the liver of raccoons from a coal fly ash site and is congruous with previous studies that found increased levels of As and Se in raccoons exposed to coal fly ash (Burger et al. 2002; Clark et al. 1989; Souza et al. 2013). Raccoons are omnivorous and likely were exposed to elevated trace element concentrations through consumption of aquatic and terrestrial vertebrates, invertebrates, and vegetation surrounding the settling ponds—organisms that have been shown to accumulate trace elements in previous studies at this site (Hopkins et al. 1998, 1999). Seasonal variation in raccoon diet (Harman and Stains 1979; Parsons et al. 2013) and precipitation can influence the availability of certain elements (Souza et al. 2013) and likely account for higher uptake of Cr, Ni, As, and Pb exhibited by raccoons captured in August compared with December.

Like other well-characterized sentinels of contaminants, raccoons appeared to be physiologically resilient to elevated trace metals in the environment. Although sample sizes were small, they were representative of the overall low levels of pathology that we found in any of the animals. The lack of significant tissue pathology or changes in body size was likely due to the relatively low levels of trace elements accumulated in raccoons in this study. Although we found significantly greater concentrations of Cu, As, Se, and Pb in animals from the contaminated site than in the reference site, the reported values were well below values associated with ex situ studies whose aims were to determine the physiological impairments resulting from toxic concentrations of Cu in sheep (1900 ppm) (Eisler 1998), As in domestic livestock (5–10 ppm) (Vreman et al. 1986), and Se in horse (44 ppm) (Eisler 1985).

While we did not find major hematological and morphological changes in our study, raccoons with an increased Cu concentration had significantly higher total abundance of helminths and *P. rara* compared with animals exposed to lower concentrations of Cu. Raccoons from the contaminated site harbored ~23% more endoparasites per raccoon than animals from the reference site due to higher abundances of *A. procyonis* and *P. rara*. The identified parasitic taxa represent a broad spectrum of transmission strategies that cannot be explained by changes in intermediate host densities alone. While *A. procyonis*, *P. rara*, and *M. ingens* have arthropod or millipede intermediate hosts (Gallati 1959; Munscher 2006), *P. lotoris* does not. Also, these differences in parasite abundance were not likely explained by differences in habitat composition (Bafundo et al. 1980; Kresta et al. 2009), because care was taken to choose contaminated and reference sites with similar habitat characteristics. In addition to increased helminth loads, we also found significant decreases in white blood cells, red blood cells, and platelets in animals with elevated levels of Se. Although it is tempting to suggest that hematological results suggested immune suppression in animals with higher trace metal accumulation, animals at both contaminated and reference sites had hematological values that were within published baseline values (Denver 2003). While it appears that parasite burden was higher in animals from the contaminated site and that the abundance was related to Cu accumulation, we did not find any relationship between WBC counts and parasite abundances. Thus, we could not make definitive inferences about the mechanisms underlying the relationship between high total helminth abundance and elevated Cu concentrations in raccoons from the contaminated site.

In situ studies of the effects of environmental contamination on biological systems are essential compliments to ex situ studies. While many ex situ studies identify lethal doses and measure morbidity due to high concentrations of trace metals, understanding the effects of sublethal, prolonged exposure in natural settings provides public health officials and wildlife managers with vital information about actual contaminant exposure and its effects. Our study constituted a novel effort that described the exposure to environmental contaminants and the physiological and parasitological status in a terrestrial host, the raccoon. We found that raccoons are sensitive sentinels of the presence of trace element contamination in the environment and were able to detect small but significant differences in trace element concentration between coal fly ash and reference sites. Raccoons from both contaminated and reference sites differed in their helminth loads and community composition. The higher exposure to contaminants did not result in deleterious health effects in raccoons, making this species a useful sentinel of trace metal bioaccumulation. Our study contributes to a growing body of literature on the role of terrestrial species and their parasites as sentinels of anthropogenic contamination and a tool for public health (Alexander et al. 2002; Miller et al. 2015). Future studies should consider the role of parasite assemblages as accumulators of trace elements and their potential relationship with the health of terrestrial hosts.

## Electronic Supplementary Material

Below is the link to the electronic supplementary material.

Supplementary material 1 (PDF 90 kb)

Supplementary material 2 (PDF 73 kb)

Supplementary material 3 (PDF 81 kb)

Supplementary material 4 (PDF 77 kb)
