# Supplementary material for: Raccoons (Procyon lotor) as Sentinels of Trace Element Contamination and Physiological Effects of Exposure to Coal Fly Ash
Source: Arch Environ Contam Toxicol. 2016 Dec 8;72(2):235–46. doi: 10.1007/s00244-016-0340-2 (PMC5281671; doi:10.1007/s00244-016-0340-2)
Supplement: Supplementary file 1 — Supplementary material 1 (PDF 90 kb) [file 244_2016_340_MOESM1_ESM.pdf]

Article title:

Raccoons (*Procyon lotor*) as sentinels of trace element contamination and physiological effects of exposure to coal fly ash

Journal name:

Archives of Environmental Contamination and Toxicology

Author names:

Felipe Hernández, Ricki E Oldenkamp, Sarah Webster, James C. Beasley, Lisa L. Farina, and Samantha M. Wisely

Affiliation and e-mail address of the corresponding author:

School of Natural Resources and Environment, University of Florida, 103 Black Hall, PO Box 116455, Gainesville, Florida 32611, USA

Department of Wildlife Ecology and Conservation, University of Florida, 110 Newins-Ziegler Hall, PO Box 110430, Gainesville, Florida 32611, USA

wisely@ufl.edu

Online Resource 1

Table A.1 List of categorical histological variables (*n* = 103) considered in the histopathology analysis of raccoons collected at two sites (contaminated (*n* = 4) and reference (*n* = 7)) in the Savanna River Site (August 2013). Categories in bold correspond to 16 variables that were found to have substantial variation in the breadth of abnormalities and were included in the multinomial logistic regression models. Binary categories (presence/absence of hepatic, ileum and colon granulomas) are also marked in bold.

**Histological variables**
Liver
**Portal fibrosis**
**Bridging fibrosis**
**Centrilobular fibrosis**
Lymphocytic portal inflammation
Plasmacytic portal inflammation
Neutrophilic portal inflammation
**Eosinophilic portal inflammation**
Lymphocytic centrilobular inflammation
Plasmacytic centrilobular inflammation
Neutrophilic centrilobular inflammation
Eosinophilic centrilobular inflammation
Lymphocytic intralobular inflammation
Plasmacytic intralobular inflammation
Neutrophilic intralobular inflammation
Eosinophilic intralobular inflammation
Histiocytic intralobular inflammation
**Granulomas presence/absence**

**Bile duct proliferation**
Hepatocyte necrosis
Large areas of necrosis
Hepatocellular lipid deposition
Hepatocellular hydropic degeneration
**Pigment in hepatocytes**
Pigment in Kupffer cells
Pigment in portal macrophages
Spleen
Lymphoid depletion
**Lymphoid hyperplasia**
**Hemosiderin**
**Reticuloendothelial hyperplasia**
Extramedullary hematopoiesis
Kidney
Proximal tubular degeneration
Proximal tubular necrosis
Lymphocytic inflammation
Plasmacytic inflammation
Neutrophilic inflammation
Eosinophilic inflammation
Histiocytic inflammation
Membranous Glomerulonephritis
Proliferative Glomerulonephritis
Membrano-proliferative Glomerulonephritis
Glomerulonephritis distribution 1
Glomerulonephritis distribution 2
Interstitial fibrosis
Tubular protein
Pigment in proximal tubular epithelium
Mineralization
Ileum
Lymphocytes in villous mucosa
Plasma cells in villous mucosa
Neutrophils in villous mucosa
Eosinophils in villous mucosa
Macrophages in villous mucosa
Lymphocytes in villous lamina propria
Plasma cells in villous lamina propria
Neutrophils in villous lamina propria
**Eosinophils in villous lamina propria**
Macrophages in villous lamina propria
Lymphocytes in deep lamina propria
**Plasma cells in deep lamina propria**
Neutrophils in deep lamina propria
**Eosinophils in deep lamina propria**

Macrophages in deep lamina propria
Lymphocytes in crypt epithelium
Plasma cells in crypt epithelium
Neutrophils in crypt epithelium
Eosinophils in crypt epithelium
Macrophages in crypt epithelium
Lymphocytes in submucosa
Plasma cells in submucosa
Neutrophils in submucosa
Eosinophils in submucosa
Macrophages in submucosa
**Granulomas presence/absence**
Crypt epithelial hyperplasia
Villous blunting
Villous fusion
Mucosal lymphangiectasia
Subucosal lymphangiectasia
**Submucosal lymphoid follicles**
Colon
Lymphocytes in superficial mucosal epithelium
Plasma cells in superficial mucosal epithelium
Neutrophils in superficial mucosal epithelium
Eosinophils in superficial mucosal epithelium
Macrophages in superficial mucosal epithelium
Lymphocytes in lamina propria
**Plasma cells in lamina propria**
Neutrophils in lamina propria
**Eosinophils in lamina propria**
Macrophages in lamina propria
Lymphocytes in gland epithelium
Plasma cells in gland epithelium
Neutrophils in gland epithelium
Eosinophils in gland epithelium
Macrophages in gland epithelium
Lymphocytes in submucosa
Plasma cells in submucosa
Neutrophils in submucosa
**Eosinophils in submucosa**
Macrophages in submucosa
**Granulomas presence/absence**
Crypt epithelial hyperplasia
Mucosal lymphangiectasia
Subucosal lymphangiectasia
Submucosal lymphoid follicles
