# Supplementary material for: Raccoons (Procyon lotor) as Sentinels of Trace Element Contamination and Physiological Effects of Exposure to Coal Fly Ash
Source: Arch Environ Contam Toxicol. 2016 Dec 8;72(2):235–46. doi: 10.1007/s00244-016-0340-2 (PMC5281671; doi:10.1007/s00244-016-0340-2)
Supplement: Supplementary file 2 — Supplementary material 2 (PDF 73 kb) [file 244_2016_340_MOESM2_ESM.pdf]

Article title:

Raccoons (*Procyon lotor*) as sentinels of trace element contamination and physiological effects of exposure to coal fly ash

Journal name:

Archives of Environmental Contamination and Toxicology

Author names:

Felipe Hernández, Ricki E Oldenkamp, Sarah Webster, James C. Beasley, Lisa L. Farina, and Samantha M. Wisely

Affiliation and e-mail address of the corresponding author:

School of Natural Resources and Environment, University of Florida, 103 Black Hall, PO Box 116455, Gainesville, Florida 32611, USA

Department of Wildlife Ecology and Conservation, University of Florida, 110 Newins-Ziegler Hall, PO Box 110430, Gainesville, Florida 32611, USA

wisely@ufl.edu

Online Resource 2

Table A.2 Pearson correlation coefficients (lower matrix) and associated *p*-values (upper matrix) for trace elements found in livers of raccoons at two sites (contaminated (n = 15) and reference (n = 11)) in the Savanna River Site (August and December 2013). Values in bold denote statistical significance (p<0.05) of the corresponding correlation coefficients between trace elements

**Trace elements**	Cu	As	Se	Pb
Cu		**0.004**	0.28	0.77
As	0.54		0.44	**<0.001**
Se	0.23	0.16		0.45
Pb	0.06	0.67	0.16	
