# Supplementary material for: Raccoons (Procyon lotor) as Sentinels of Trace Element Contamination and Physiological Effects of Exposure to Coal Fly Ash
Source: Arch Environ Contam Toxicol. 2016 Dec 8;72(2):235–46. doi: 10.1007/s00244-016-0340-2 (PMC5281671; doi:10.1007/s00244-016-0340-2)
Supplement: Supplementary file 3 — Supplementary material 3 (PDF 81 kb) [file 244_2016_340_MOESM3_ESM.pdf]

Article title:

Raccoons (*Procyon lotor*) as sentinels of trace element contamination and physiological effects of exposure to coal fly ash

Journal name:

Archives of Environmental Contamination and Toxicology

Author names:

Felipe Hernández, Ricki E Oldenkamp, Sarah Webster, James C. Beasley, Lisa L. Farina, and Samantha M. Wisely

Affiliation and e-mail address of the corresponding author:

School of Natural Resources and Environment, University of Florida, 103 Black Hall, PO Box 116455, Gainesville, Florida 32611, USA

Department of Wildlife Ecology and Conservation, University of Florida, 110 Newins-Ziegler Hall, PO Box 110430, Gainesville, Florida 32611, USA

wisely@ufl.edu

Online Resource 3

Table A.3 Mean±S.E. (range) of values of morphometry of raccoons from contaminated and reference sites in the SRS (August and December 2013)

Morphometry	**Sites**					
	Contaminated			Reference		
	**Male**	**Female**	**Range**	**Male**	**Female**	**Range**
n	9	6		10	1	
Body mass (kg)	6.0±0.4	4.1±0.4	(3.0-8.6)	5.5±0.4	3.3	(3.3-7.7)
Nose-anus length (cm)	59.4±1.2	50.2±1.4	(47.0-66.3)	59.2±0.9	49.6	(49.6-62.2)
Nose-anus length/weight	0.1±0.0	0.1±0.0	(0.06-0.13)	0.1±0.0	0.07	(0.07-0.13)
Tail (cm)	25.1±0.5	23.8±0.7	(20.9-27.4)	25.5±0.7	26	(21.7-28.6)
Left hind foot (cm)	10.8±0.3	9.4±0.4	(8.1-11.8)	10.7±0.1	10	(9.9-11.3)
Left ear (cm)	6.0±0.2	5.4±0.3	(4.3-6.8)	6.1±0.1	6.3	(5.3-6.8)
Chest (cm)	37.9±1.2	32.4±1.5	(29.3-44.5)	37.6±1.1	32.9	(32.2-43.9)
