# Supplementary material for: Raccoons (Procyon lotor) as Sentinels of Trace Element Contamination and Physiological Effects of Exposure to Coal Fly Ash
Source: Arch Environ Contam Toxicol. 2016 Dec 8;72(2):235–46. doi: 10.1007/s00244-016-0340-2 (PMC5281671; doi:10.1007/s00244-016-0340-2)
Supplement: Supplementary file 4 — Supplementary material 4 (PDF 77 kb) [file 244_2016_340_MOESM4_ESM.pdf]

Article title:

Raccoons (*Procyon lotor*) as sentinels of trace element contamination and physiological effects of exposure to coal fly ash

Journal name:

Archives of Environmental Contamination and Toxicology

Author names:

Felipe Hernández, Ricki E Oldenkamp, Sarah Webster, James C. Beasley, Lisa L. Farina, and Samantha M. Wisely

Affiliation and e-mail address of the corresponding author:

School of Natural Resources and Environment, University of Florida, 103 Black Hall, PO Box 116455, Gainesville, Florida 32611, USA

Department of Wildlife Ecology and Conservation, University of Florida, 110 Newins-Ziegler Hall, PO Box 110430, Gainesville, Florida 32611, USA

wisely@ufl.edu

Online Resource 4

Table A.4 Principal Components Analysis (PCA) values on morphometic measurements of raccoons from contaminated and reference sites (n = 26) in the SRS (August and December 2013)

Component	*PC loadings (eigenvectors)*
MorphPC1[a,b]	
PC variables	
Body mass	-0.1923
Nose-anus length	-0.7749
Weight/nose-anus length ratio	-0.0024
Tail	-0.0621
Left hindfoot	-0.0952
Left ear	-0.0359
Chest	-0.5901

[a]Eigenvalue: 41.31

[b]Cumulative % variance: 82%
